# Canagliflozin for Prevention of Cardiovascular and Renal Outcomes in type2 Diabetes: A Systematic Review and Meta-analysis of Randomized Controlled Trials

**DOI:** 10.3389/fphar.2021.691878

**Published:** 2021-07-19

**Authors:** Lei Tian, Yuzi Cai, Huijuan Zheng, Sinan Ai, Mengqi Zhou, Qian Luo, Jingyi Tang, Weijing Liu, Yaoxian Wang

**Affiliations:** Renal Research Institution of Beijing University of Chinese Medicine, and Key Laboratory of Chinese Internal Medicine of Ministry of Education and Beijing, Dongzhimen Hospital Affiliated to Beijing University of Chinese Medicine, Beijing University of Chinese Medicine, Beijing, China

**Keywords:** canagliflozin, Type 2 diabetes mellitus, Cardiovascular outcomes, renal function, renal events

## Abstract

**Objective:** We aimed to evaluate the efficacy of canagliflozin for the treatment of specific cardiovascular and renal outcomes in Type 2 diabetes mellitus (T2DM) patients by means of a systematic review and meta-analysis.

**Methods:** We performed comprehensive searches of PubMed, the Cochrane Library, and Embase for randomized, placebo-controlled trials of the treatment of T2DM with canagliflozin that were published to 28 September 2020. The cardiovascular outcomes recorded were cardiovascular mortality, heart failure, myocardial infarction, and stroke. The renal composite outcomes recorded were end-stage renal disease (ESRD), renal death. The data for the principal cardiovascular outcomes, ESRD, and renal death were pooled and expressed as Hazard ratios (HRs) with 95% confidence intervals (CIs). Two reviewers independently selected the trials and extracted the data.

**Results:** We identified a total of 1,741 publications, leaving 96 for their titles, abstracts and full-text review. Of these, 10 trials met the inclusion criteria and were finally included in our meta-analysis. The meta-analysis showed that canagliflozin significantly reduced the risk of heart failure in T2DM by 36% (HR 0.64, 95% CI 0.53 to 0.77, p = 0.000). The effects of canagliflozin on non-fatal myocardial infarction or non-fatal stroke (HR 0.84, 95% CI: 0.76 to 0.93, p = 0.001), cardiovascular mortality (HR 0.84, 95% CI 0.72 to 0.97, p = 0.021), and myocardial infarction (HR 0.84, 95% CI 0.70 to 1.00, p = 0.045) in patients with T2DM were relatively small, reducing the risks by 16%. In addition, canagliflozin reduced the risk of stroke in T2DM patients by 13% (HR 0.87, 95% CI 0.71 to 1.06, p = 0.166). Moreover, canagliflozin significantly reduced the risk of the composite renal event of ESRD or renal death by 36% (HR 0.64, 95% CI 0.54 to 0.75, p = 0.000).

**Conclusion:** This meta-analysis suggests that canagliflozin protects against cardiovascular and renal outcomes in patients with T2DM.

**Systematic Review Registration**: [https://www.crd.york.ac.uk/prospero], identifier [CRD42020210315]

## Introduction

Type 2 diabetes mellitus (T2DM) consists of a set of metabolic defects and is principally characterized by relative insulin deficiency resulting from a combination of pancreatic β-cell dysfunction and insulin resistance in target organs (Chatterjee et al., 2017). More than 463 million people worldwide were estimated to have diabetes mellitus in 2019, and this number is predicted to rise to ∼693 million by 2045 (https://diabetesatlas.org/en/). The prevalence of T2DM is rising rapidly all over the world, and patients with this form account for ∼90% of the total (Mannino et al., 2019). As a consequence, T2DM has become a major threat to health worldwide.

The excess morbidity and mortality associated with T2DM are mainly the result of cardiovascular and renal complications (Braunwald, 2019). Patients with diabetes are at a higher risk of major adverse cardiovascular events, which include myocardial infarction, heart failure, stroke, and cardiovascular mortality. A previous retrospective cohort study showed that individuals with T2DM develop cardiovascular disease 14.6 years earlier than those without T2DM, and it tends to be more serious (Booth et al., 2006). Renal disease is another common complication of T2DM, and approximately 10% of the deaths of patients with T2DM are related to renal failure (Van Dieren et al., 2010). In China, diabetes-related chronic kidney disease was the primary cause of end-stage renal disease (ESRD) in the general population in 2010 (Zhang et al., 2016). Therefore, when selecting antidiabetic drugs for patients with T2DM, physicians should consider their efficacy for the prevention or treatment of cardiovascular and renal outcomes.

Sodium-glucose cotransporter-2 inhibitors (SGLT2is) represent a novel category of glucose-lowering agent that increase natriuresis, cause intravascular volume contraction, and change intrarenal hemodynamics, which likely contributes to their beneficial effects on blood pressure, glycemia, intrarenal hemodynamics, and albuminuria (Heerspink et al., 2016; Wu et al., 2016). These pleiotropic effects of SGLT2is have been shown to help prevent cardiovascular events and preserve the renal function of T2DM patients in several large, well-conducted randomized controlled trials (RCTs) (Wanner et al., 2016; Kosiborod et al., 2017; Perkovic et al., 2019). Previous meta-analyses have evaluated the effects of SGLT2is on cardiovascular and renal outcomes in T2DM (Zhang et al., 2018; Zelniker et al., 2019a; Bae et al., 2019; Zelniker et al., 2019b). However, canagliflozin, which was the first SGLT2i to be approved by the U.S. Food and Drug Administration for the therapy of adult T2DM patients, was used in a small proportion of the studies included in these meta-analyses: in those by Zelniker et al. (2019b) and Zhang et al. (2018), only one RCT of canagliflozin was included. Moreover, Several previous meta-analyses have determined the effects of canagliflozin on other aspects of T2DM. The meta-analysis by Blonde et al. showed that canagliflozin treatment helps T2DM patients achieve their recommended glycemic, body mass, blood pressure, and high-density lipoprotein-cholesterol targets (Blonde et al., 2015). In addition, Yang et al. found evidence to suggest that treatment with canagliflozin reduces glycated hemoglobin (HbA1c) in patients with T2DM, but they considered that there was insufficient evidence regarding the effect of canagliflozin on the incidence of cardiovascular events in T2DM to draw conclusions (Yang et al., 2014). Thus, there has been no systematic review or meta-analysis of the effects of canagliflozin on the incidences of cardiovascular events or renal events in patients with T2DM. A recent, multicenter international clinical trial including 4,401 participants named Canagliflozin and Renal Endpoints in Diabetes with Established Nephropathy Clinical Evaluation (CREDENCE), which as gold standard phase III study, showed that canagliflozin significantly reduced major cardiovascular events and renal failure in patients with T2DM and chronic kidney disease (Perkovic et al., 2019). Previous studies have also shown that Canaglifozin increases the risk of adverse renal events and acute kidney injury in patients with predisposing factors such as hypoglycemia, chronic kidney disease (CKD), heart failure, and potentially nephrotoxic drugs (Vasilakou et al., 2013; Tang et al., 2017). Therefore, concern has been raised in terms of the effect of canagliflozin on renal function (Oshima et al., 2021). Moreover, whether the effects of canagliflozin on renal function are dose-dependent remains uncertain. In order to support the appropriate use of canagliflozin in clinical practice for patients with T2DM, we performed this systematic review and meta-analysis of randomized controlled trials (RCTs) to evaluate the effects of canagliflozin on specific clinical cardiovascular and renal outcomes, and further analyzed the effects of different doses of canagliflozin, including 100 mg/ day and 300 mg/ day, on the renal function of patients with T2DM.

### Patients and Methods

We performed a systematic review and meta-analysis according to the preferred reporting items for systematic review and meta-analyses (PRISMA) guidelines. The systematic review protocol has been registered in the PROSPERO database (International Prospective Register of Systematic Reviews, https://www.crd.york.ac.uk/prospero; registration number CRD42020210315).

### Data Sources and Searches

We performed comprehensive searches of PubMed, the Cochrane Library, and Embase from their inception to September 2020, using the search terms “canagliflozin”, “type 2 diabetes”, and related words. We also manually screened the references of the selected studies and related meta-analyses and review articles to identify eligible trials that were not found using the database searches. The searches were limited to English-language articles. The selected documents were edited and managed using a bibliographic database created in EndNote X8, and duplicate documents were removed.

### Study Inclusion and Exclusion Criteria

We screened studies on the basis of the following inclusion criteria. 1) They were RCTs that compared canagliflozin with placebo or other glucose-lowering treatments. 2) The participants were male or female patients with T2DM who were ≥18 years old, and had HbA1c values of between 6.5 and 10.5%. 3) The duration of intervention was ≥13 weeks 4) The outcomes of the study included at least one cardiovascular or renal event, or renal function (including estimated glomerular filtration rate (eGFR) and Creatinine). The exclusion criteria were as follows. 1) The participants had type 1 diabetes mellitus or a history of hereditary glucose or galactose malabsorption. 2) The study did not specify the inclusion or exclusion criteria. 3) Preclinical research in animal models. 4) Repeated use of data for secondary analyses.

### Data Extraction and Quality Assessment

Two reviewers independently conducted preliminary screens of the title and/or abstract of each candidate article, and if there was insufficient information regarding the inclusion or exclusion criteria, then the full text of the article was evaluated. For RCTs that satisfied the inclusion criteria, the basic features of the trial, including the investigators, year, country, sample size, the average age of the participants at baseline, the dose of canagliflozin, duration of the intervention, and participant HbA1c were collected. The outcomes mainly comprised renal function; renal adverse events; cardiovascular events (myocardial infarction, heart failure, stroke, and cardiovascular mortality); cardiovascular mortality; composites of renal function, including estimated glomerular filtration rate (eGFR) and creatinine, and standardized renal events, including ESRD or renal mortality.

The methodological quality and risk of bias for the included trials were evaluated using the Cochrane Collaboration’s risk of bias tool, which included randomization, quality of blinding, allocation concealment, and reporting bias categories. For each category, the trial was graded as high, or low or unclear. Two reviewers independently performed the data extraction and quality evaluation, and if there were any disagreements they were resolved by discussion. The analyses were performed using Review Manager 5.2 (Cochrane Collaboration, http://www.cochrane.org).

### Data Synthesis and Analysis

Continuous data are expressed as mean difference ± SD. Standardized mean differences (SMD) were calculated as a summary statistic. In addition, we calculated pooled hazard ratios (HRs) with 95% confidence intervals (CIs) for the effects of canagliflozin on the incidences of cardiovascular and renal events. Statistical analyses were performed using Stata MP Software version 14. *p* < 0.05 was regarded as indicating a statistically significant difference.

We evaluated heterogeneity using the Cochrane Q statistic and report it as I^2^. Heterogeneity was regarded as low if I^2^ ≤ 40%, moderate if 40–70%, and high if >70%. If the *p*-value was ≥0.1 and the I^2^ was ≤40%, heterogeneity was present among the included trials and a fixed-effect model was used, and if not, we used a random-effect model. When there was heterogeneity in the included trials, the following methods were used to identify the source of the heterogeneity. A) Subgroup analysis was performed according to the sex, age, country, canagliflozin dose, drug category, duration of follow-up, and disease classification of the trial. B) As a sensitivity analysis, RCTs were eliminated one by one to identify which were responsible for the heterogeneity.

The Egger test was used to identify any publication bias, in Stata MP software version 14.0. When a result of *p* < 0.05 was obtained, this indicated the possibility of publication bias.

## Results

### Description of Included Trials

**FIGURE 1:**
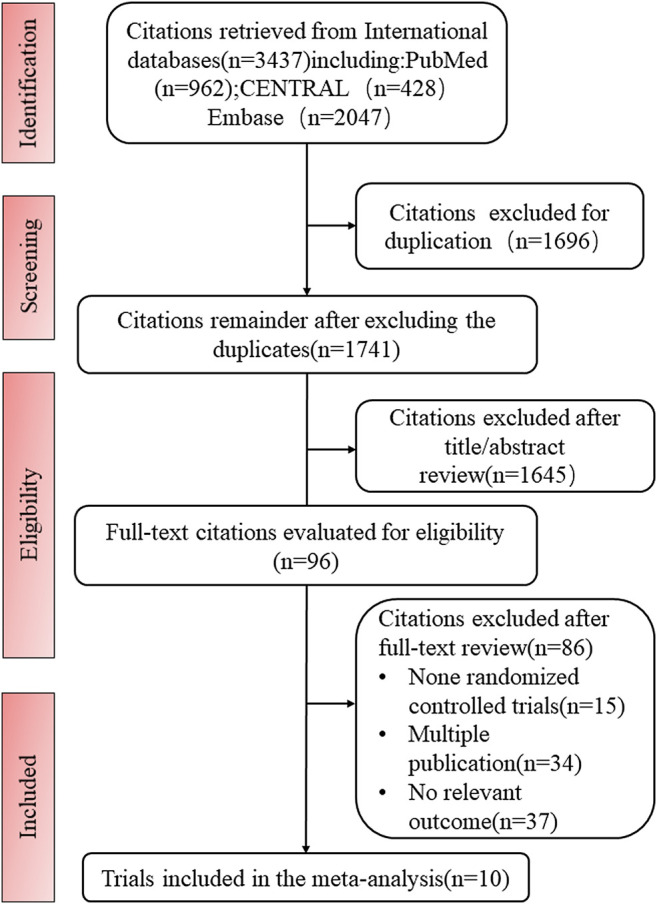
PRISMA flow diagram of study selection process.

We identified a total of 3,437 publications during the searches. Of these, 1741 publications remained after the exclusion of duplicates, but a further 1,645 were excluded after review of their titles and abstracts, leaving 96 for full-text review. Of these, 10 trials met the inclusion criteria (Figure 1).

The characteristics of the included trials are presented in Table 1 Bode 2015 (Bode et al., 2015), CANVAS 2017 (Neal et al., 2017), CANVAS-R 2017 (Neal et al., 2017), CREDENCE 2019 (Perkovic et al., 2019), Forst 2014 (Forst et al., 2014), Gonzalez 2013 (Lavalle-González et al., 2013), Ji 2015 (Ji et al., 2015), Stenlof 2013 (Stenlöf et al., 2013), Wilding 2013 (Wilding et al., 2013), Yale 2014 (Yale et al., 2014), Qiu 2014 (Qiu et al., 2014). In total, 19,363 patients with T2DM were randomly assigned to canagliflozin treatment or a comparator (placebo or other glucose-lowering treatment). The ages of the participants were between 18 and 80^ ^years. The sample sizes of the trials ranged from 269 to 10,142, and the duration of follow-up ranged from 13 to 104 weeks. The baseline HbA1c was 6.5–10.5%. Of the 10 trials, the CREDENCE trial and Yale’s study enrolled patients who had both T2DM and nephropathy. In addition, the CANVAS program consisted of two multi-center RCTs, CANVAS, and CANVAS-R, which were conducted in parallel. The CANVAS and CREDENCE programs aimed to evaluate the effect of canagliflozin treatment on cardiovascular and renal outcomes in patients with T2DM and a history or high risk of cardiovascular disease. The remaining nine studies evaluated the efficacy (including kidney function) and safety of canagliflozin in participants with T2DM. All the included trials were published in English between 2013 and 2020.

**TABLE 1:** Characteristics of included randomized controlled trials.

*Study (reference number)*	*Country*	*N (Case: Control)*	*Type of study*	*Administered Canagliflozin Dose*	*Subject type*	*% female*	*Duration in weeks*	*Age (years)*	*Glycated hemoglobin level*	*Main outcome measures*
B Bode, et al. (2015)	USA	714 (477/237)	Randomized, double-blind, parallel-group, placebo-controlled trail	100 mg/d;300 mg/d	type 2 diabetes mellitus	318 (44.5%)	104	50–80 years	HbA1c≥7.0 to≤10.0%	Glycaemic Efficacy Endpoints. Safety and Tolerability.
Neal et al. (2017) (CANVAS)	Australian	10142 (5795/4347)	Randomize, double-blind, parallel-group, placebo-controlled trail	100 mg/d;300 mg/d	type 2 diabetes mellitus	3633 (35.8)	13	More than 30 years old(mena age=63.3	≥7.0% and ≤10.5%	Cardiovascular Risk; Cardiovascular Outcomes, Death, and Hospitalizations; Renal Outcomes; Safety Outcomes
Perkovic et al. (2019) (CREDENCE)	Australian	4401 (2202/2199)	Randomize, double-blind, parallel-group, placebo-controlled trail	100 mg/d;	DKD	1494 (33.9)	13	at least 30 years of age	6.5 to 10.5% in Germany, according to a country amendment	Renal components Safety Outcomes; Cardiovascular Risk
T. Forst (2014)	Germany	342 (113/114/115)	Randomize, double-blind, parallel-group, PBO/SITA-controlled trail	100 mg/d;300 mg/d	type 2 diabetes mellitus	126 (36.8)	52	aged ≥18 and ≤ 80 years	≥7.0% to ≤10.5%	Efficacy: Glycaemic Efficacy Endpoints. Body Weight, BP and Lipids, β-Cell Function; Safety and Tolerability
Lavalle-Gonzalez et al. (2013)	Mexico	1284 (368/367/183/366)	Randomize, double-blind, parallel-group, PBO/SITA-controlled trail	100 mg/d;300 mg/d	type 2 diabetes mellitus	679 (52.9)	52	aged ≥18 and ≤80 years	≥7.0% and ≤10.5%	Effect on glycaemic variables; Effect on body weight, BP and lipids; Safety and tolerability
Lining Ji, et al. (2015)	China	678 (223/227/226)	Randomize, double-blind, parallel-group, placebo-controlled trail	100 mg/d;300 mg/d	type 2 diabetes mellitus	114 (50.2)	18	≥18 and ≤80 years	(HbA1c) ≥7.0 and ≤10.5%	Efficacy: Glycaemic Efficacy Endpoints; body Weight and BP; Lipids; Safety
K.Stenlof (2013)	Sweden	584 (195/197/192)	Randomize, double-blind, parallel-group, placebo-controlled trail	100 mg/d;300 mg/d	type 2 diabetes mellitus	326 (55.8%)	26	18–80 years of age	≥7.0 and ≤10.0%	High Glycaemic Substudy; Glycaemic Efficacy Endpoints; Body Weight, BP and Lipids; β-cell Function; High Glycaemic Substudy
J.P.H Wilding, et al. (2013)	UK	469 (157/156/156)	Randomize, double-blind, parallel-group, placebo-controlled trail	100 mg/d;300 mg/d	type 2 diabetes mellitus	230 (49.0)	52	18-80 years	HbA1c≥7.0 and ≤10.5%	Efficacy: Glycaemic Efficacy Endpoints; Other efficacy end-points; Safety and tolerability
J-F Yale (2014)	Canada	269 (90/90/89)	Randomize, double-blind, parallel-group, placebo-controlled trail	100 mg/d;300 mg/d	type 2 diabetes mellitus	106 (39.4)	52	≥25 years of age	≥7.0 and ≤10.5%	Overall Safety and Tolerability. Measures of Renal Function.
Rong Qiu et al. (2014)	USA	480 (93/93/93)	Randomize, double-blind, placebo-controlled trail.	100 mg/d;300 mg/d	type 2 diabetes mellitus	149 (53.4)	18	Aged 18 to 80 years	≥7.0 and ≤10.5%	Efficacy: Glycaemic parameters; Body weight, and lipids; Safety

BP, blood pressure

### Quality Assessment of Individual Trials

**FIGURE 2:**
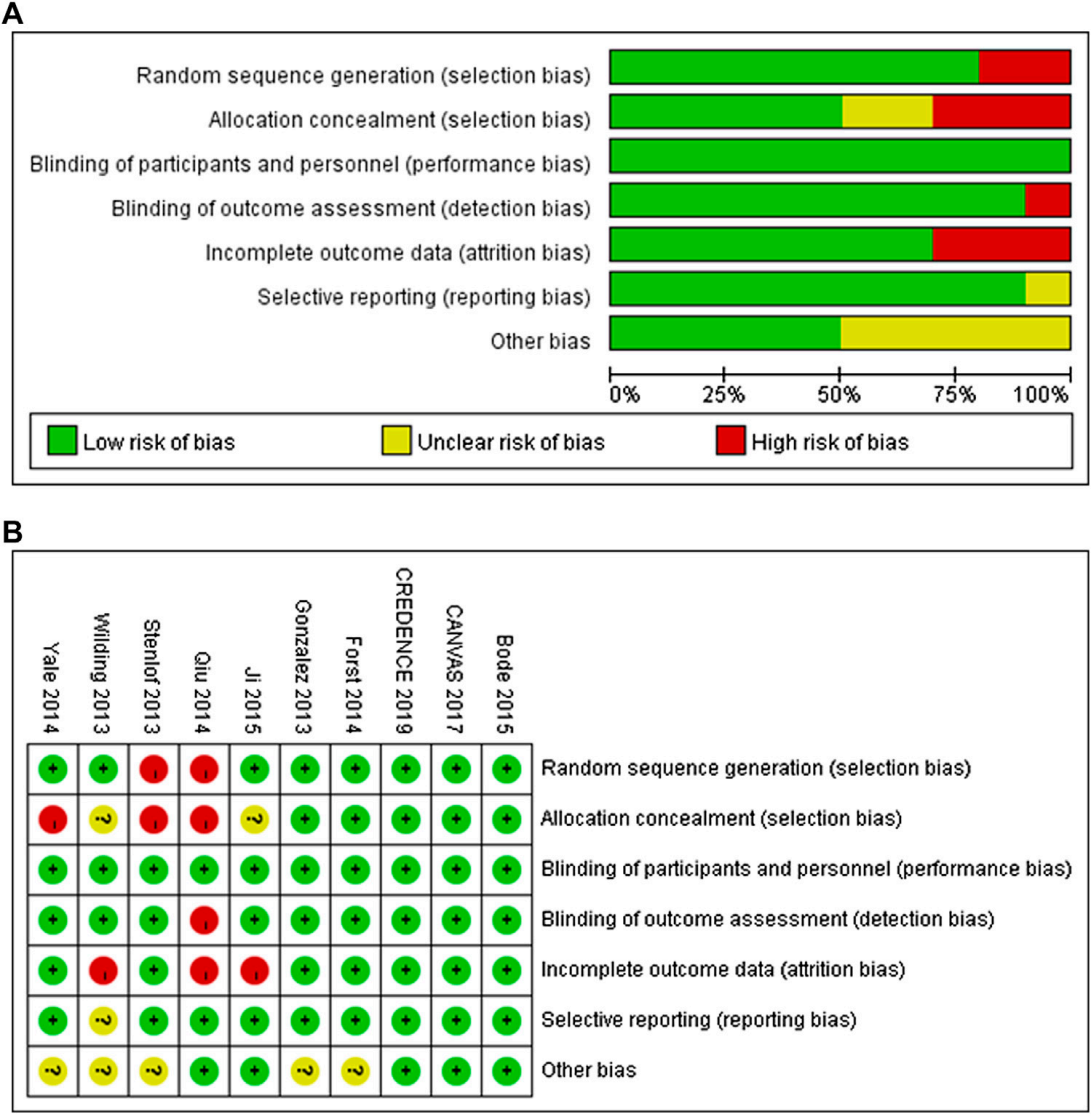
Risk of bias graph **(A)** and risk of bias summary **(B)** for included RCTs.

A summary of the risks of bias for the included studies, assessed using the Cochrane Collaboration tool, is presented in Figures 2A,B. Eight trials were reported to have used randomized sequence generation, but the methods used in the other two trials were not clearly stated. Regarding allocation concealment, five trials were low risk, three trials were high risk, and for two trials this was unclear. All the trials had involved blinding of the participants and investigators. With respect to detection bias, there was a lack of clarity for one trial, but the others were categorized as low risk. Furthermore, all of the trials except one were assessed to have a low risk of reporting bias.

### Effect of Canagliflozin on Cardiovascular Outcomes

**FIGURE 3:**
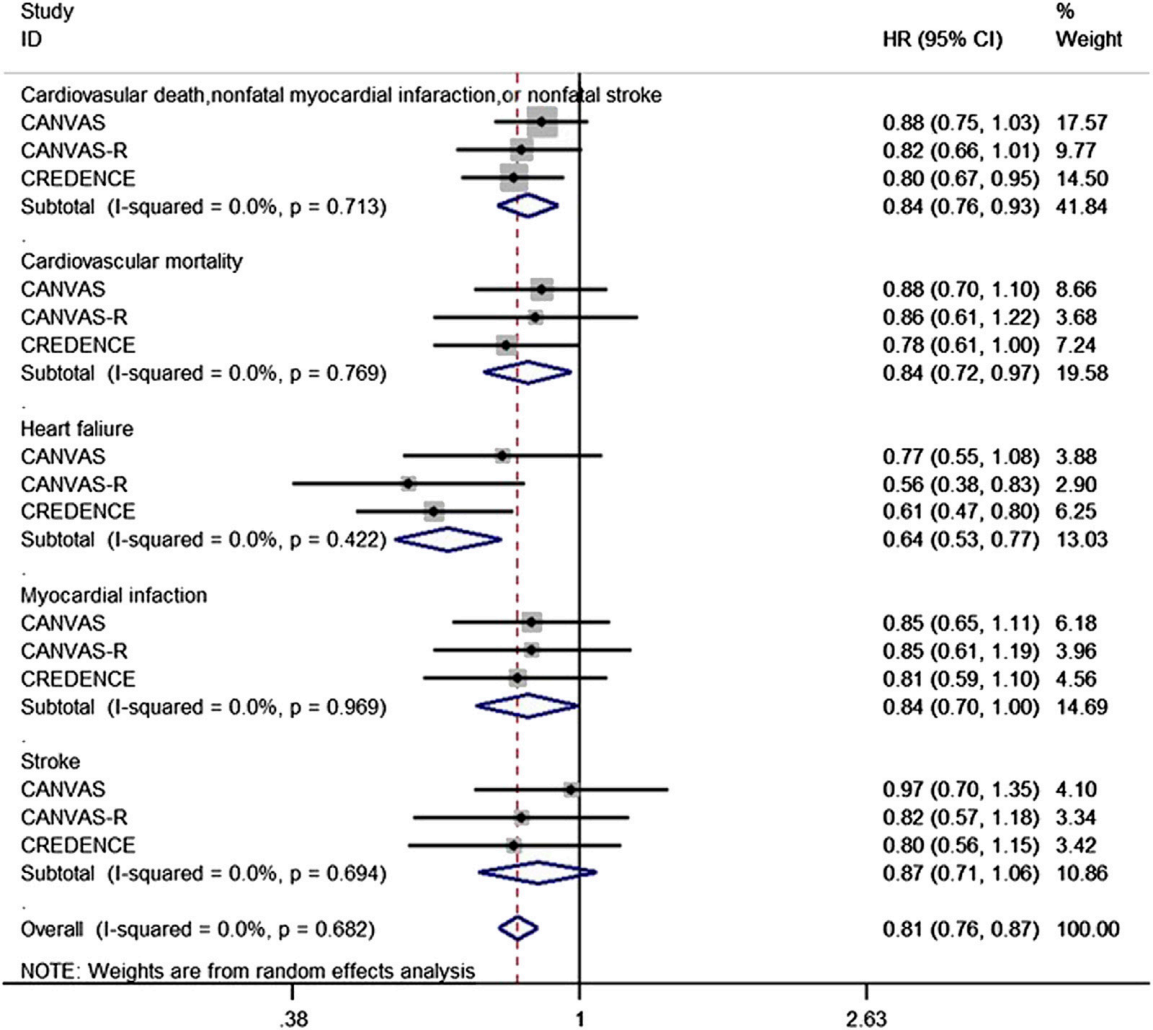
Forest plot of the effect of canagliflozin on the composite of cardiovascular death, cardiovascular mortality, heart failure, Myocardial infaction and stroke of the T2DM participants.

We identified a total of 14,543 participants in two independent trials (the CANVAS and CREDENCE programs, with the former including the parallel CANVAS and CANVAS-R trials) that were eligible for inclusion. The publications principally reported a cardiovascular composite outcome that consisted of cardiovascular mortality, non-fatal myocardial infarction or non-fatal stroke, heart failure, myocardial infarction, and stroke. The present analysis showed that canagliflozin significantly reduced the risk of heart failure in T2DM by 36% (HR 0.64, 95% CI 0.53–0.77, *p* = 0.000). However, the effect of canagliflozin on non-fatal myocardial infarction or non-fatal stroke (HR 0.84, 95% CI 0.76–0.93, *p* = 0.001) and myocardial infarction (HR 0.84, 95% CI 0.70–1.00, *p* = 0.045) in patients with T2DM was limited to a 16% reduction in risk. Moreover, the risk of cardiovascular mortality of T2DM patients was reduced by 16% (HR 0.84, 95% CI 0.72–0.97, *p* = 0.021). In addition, canagliflozin only reduced the risk of stroke in patients with T2DM by 13% (HR 0.87, 95% CI 0.71–1.06, *p* = 0.166). There was no obvious evidence of heterogeneity in the data derived from the two independent programs with respect to any of the cardiovascular events reported (I^2^ = 0.0%, *p* > 0.1) (Figure 3A).

### Effect of Canagliflozin on Clinical Renal Outcomes

**FIGURE 4:**
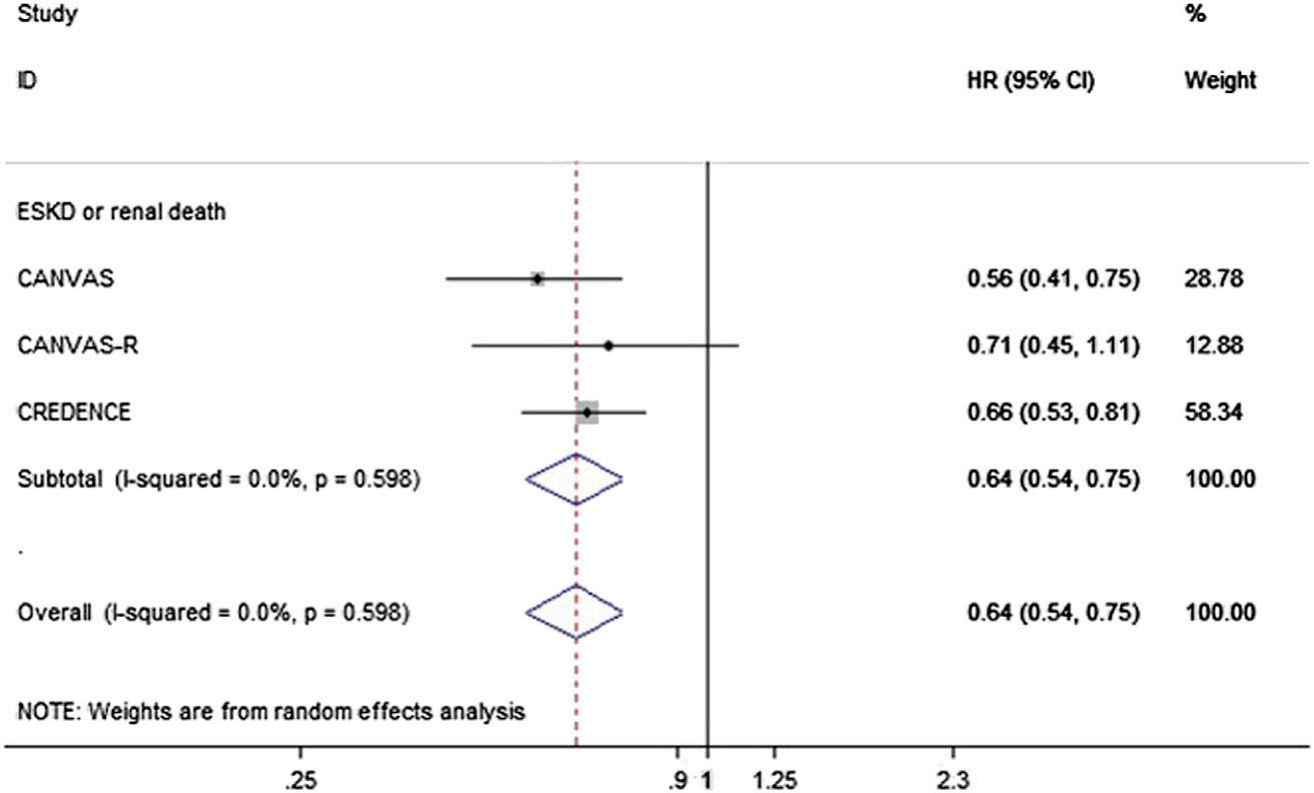
Forest plot of the effects of canagliflozin on the composite of ESKD or renal death of the T2DM participants.

Data regarding the effects of canagliflozin on ESRD and renal mortality were obtained from articles reporting the results of the CANVAS CANVAS-R and CREDENCE programs, which contained up to 14,543 patients with T2DM. In these programs, canagliflozin reduced the risk of the composite renal endpoint of ESRD or renal mortality by 36% (HR 0.64, 95% CI 0.54 to 0.75, *p* = 0.000), with no evidence of heterogeneity between the two programs (Figure 4A).

### Effect of Cangliflozin on Laboratory Changes of Renal Function

**FIGURE 5:**
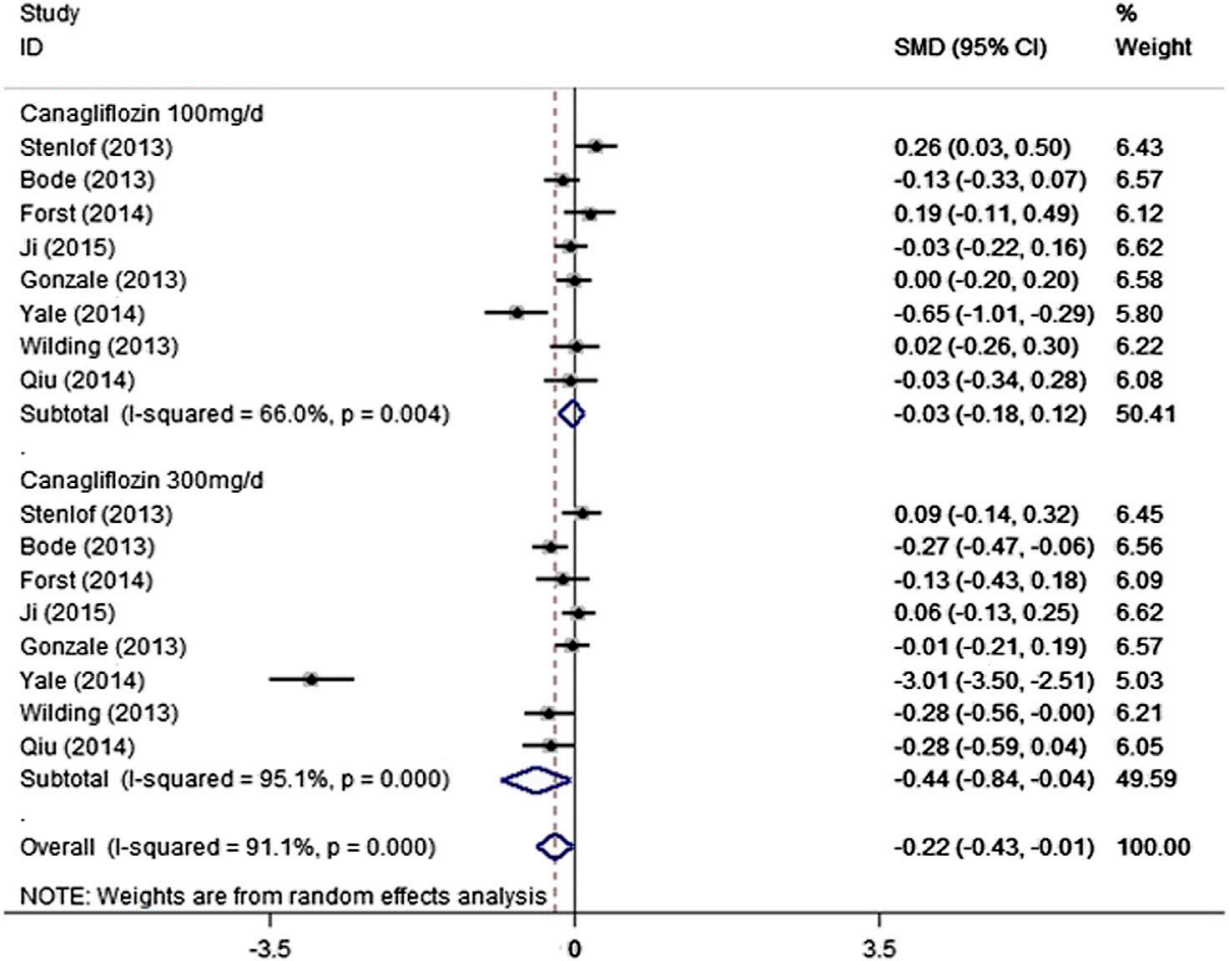
Forest plot detailing subgroup analysis of the effect of canagliflozin on the eGFR of the T2DM participants based on dosage.

**FIGURE 6:**
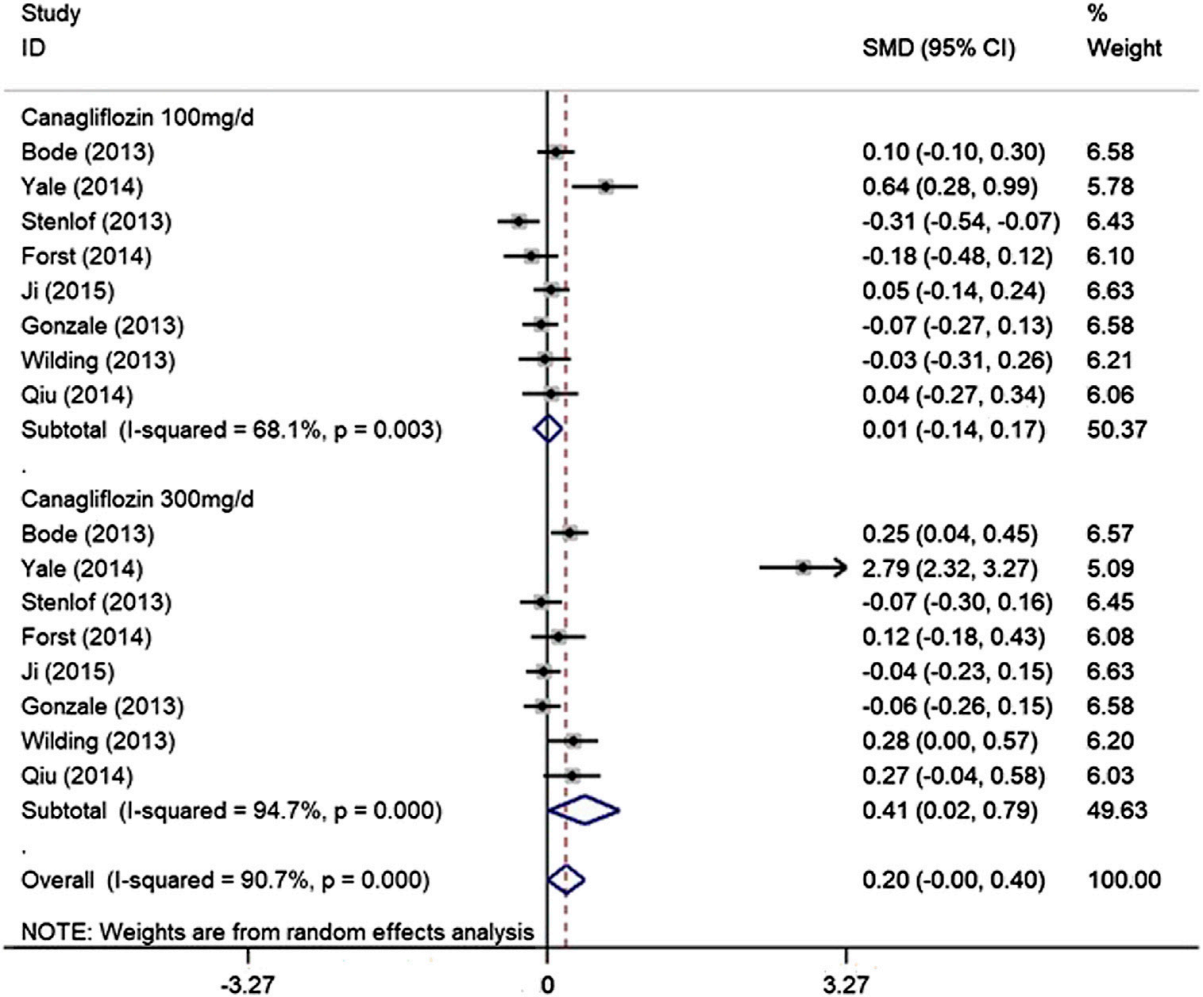
Forest plot detailing subgroup analysis of the effect of canagliflozin on the Creatinine of the T2DM participants based on dosage.

Our analysis showed that 100 mg/day canagliflozin had no effect on eGFR, in comparison to the control group (SMD − 0.03, 95% CI − 0.18 to 0.12, *p* = 0.703). When the dose used was 300 mg/day, the results of the analysis indicated that canagliflozin reduced eGFR in participants with T2DM, compared with the control group (SMD − 0.44, 95% CI − 0.84 to − 0.04, *p* = 0.033). However, there was notable heterogeneity among the included trials (I^2^ = 95.1%, *p* = 0.000) (Figure 5A). During the sensitivity analysis, elimination of the data of Yale et al. (2014) reduced the heterogeneity to 23.4% (100 mg/ day canagliflozin) and 47.7% (300 mg/ day canagliflozin) (Supplementary Figure S1A,B) and affected the overall outcome reflected in 300 mg canagliflozin could not significantly reduce eGFR. Furthermore, after deleting Yale’s data, there was no evidence of publication bias among the trials (Egger’s test*, p* = 0.769). In addition, our analysis indicated that 100 mg/ day canagliflozin did not significantly change the T2DM patinent’s serum creatinine level (SMD 0.01, 95% CI − 0.14 to 0.17, *p* = 0.883), but 300 mg/day canagliflozin increased the creatinine *vs*. the control group (SMD 0.41, 95% CI 0.02 to 0.79, *p* = 0.039) (Figure 6A). However, there was a high level of heterogeneity among the studies. In the sensitivity analysis, when the study by Yale et al. (2014) was excluded, the heterogeneity was reduced (100 mg/day canagliflozin: I^2^ = 32.9%, *p* = 0.177; 300 mg/day canagliflozin: I^2^ = 45%, *p* = 0.091) (Supplementary Figure S2A,B). Furthermore, after the deletion of the data obtained by Yale et al. (2014), fixed-effect model analysis indicated that neither 100 mg/ day canagliflozin (SMD − 0.04, 95% CI − 0.13 to 0.05, *p* = 0.345) (Supplementary Figure S2A) nor 300 mg/day canagliflozin (SMD 0.08, 95% CI − 0.01 to 0.16, *p* = 0.092) (Supplementary Figure S2B) had reduced the level of creatinine concentration in patients with T2DM.Moreover, after deleting Yale’s data, there was no evidence of publication bias among the trials (Egger’s test,*p* = 0.657).

## Discussion

The most common complications of T2DM are cardiovascular and renal, and these are the main causes of disability and death. Canagliflozin is an SGLT2 inhibitor, a representative of a widely used class of anti-hyperglycemic agents. Although there have been some previous studies of the efficacy and safety of the use of canagliflozin in patients with T2DM, few meta-analyses have been performed to evaluate the effect of canagliflozin on clinical cardiovascular and renal outcome in patients with T2DM. To our knowledge, this study is the first meta-analysis of RCTs to systematically assess whether canagliflozin has notable effects on cardiovascular and renal outcomes in patients with T2DM. In addition, understanding the effects of long-term administration of different doses of canagliflozin on the renal function of patients with T2DM is important to guide the better use of canagliflozin in clinical practice. The analysis showed that canagliflozin improves cardiovascular outcomes and significantly reduced the risk of ESRD and renal death in patients with T2DM. Moreover, there was no difference in the overall changes of eGFR and creatinine for the groups whether they took 100 mg/ day or 300 mg/ day of canagliflozin compared to placebo. In addition, the effect of canagliflozin on eGFR and creatinine might affect by the baseline eGFR.

In terms of cardiovascular outcomes, our findings are consistent with those of studies of other SGLT2is. For example, Wiviott et al. showed that treatment with dapagliflozin reduces the incidence of HHF and cardiovascular mortality (Wiviott et al., 2019), and Zinman et al. found that the treatment of patients with T2DM who are high risk for cardiovascular events with empagliflozin is associated with lower incidences of major cardiovascular complications and all-cause mortality (Zinman et al., 2015).

The low level of natriuresis and the presence of hypertension implicate sodium retention and vascular volume expansion as being pivotal in cardiovascular outcomes, and in particular heart failure (Sattar and Mcguire, 2018). An increase in urinary sodium excretion and vascular contraction are possible mechanisms for the effect of canagliflozin to reduce blood pressure. In addition, preclinical data have suggested that canagliflozin might reduce infarct size (Lim et al., 2019), delay the progression of atherosclerosis, and downregulate the expression of adhesion molecules and inflammatory molecules, including vascular cell adhesion molecule-1 and monocyte chemotaxis protein-1 (Nasiri-Ansari et al., 2018). Moreover, the unique hypoglycemic mechanism of SGLT2is, which involves an increase in glucosuria and is not insulin-dependent, means that the risk of hypoglycemia is low (Abdul-Ghani et al., 2011), which is conducive to lower cardiovascular risk. Furthermore, SGLT2is shift metabolism from carbohydrate to lipid use, causing a degree of ketogenesis, which may provide an alternative energy substrate for cardiomyocytes under conditions of ischemic stress (Ferrannini et al., 2016). Thus, there is a mechanistic basis for the beneficial effects of canagliflozin on cardiovascular outcomes in patients with T2DM.

The present analysis suggests that canagliflozin reduces the risks of ESRD and renal death in patients with T2DM. In the CREDENCE study, which assessed the primary composite renal outcome, canagliflozin indicated 32% of risk reduction of ESRD compared with placebo in patients with T2DM, and 30% of risk reduction of doubling of serum creatinine and renal death (Perkovic et al., 2019). The mechanism of potential renal outcomes protection of canagliflozin has not been fully explained. The study of 2 years of follow-up data from RCTs indicates that the beneficial effects of canagliflozin on renal outcomes are unlikely to be attributable to its modest effect on circulating glucose concentration. Published data suggest that the renoprotective effect of SGLT2is occurs within 3.1 years and that the reduction in HbA1c is mild ( − 0.28%), which further implies that an improvement in glycemic control is unlikely to explain the beneficial effects of SGLT2 inhibition on renal outcomes. Instead, the nephroprotective effect is most likely to related to the changes in intrarenal hemodynamics (Defronzo et al., 2017). Previous studies also indicated that anticipated 5 mm Hg lowering of systolic blood pressure could result in a reduction for about 10% in the risk for developing renal endpoints. The beneficial effects of canagliflozin on clinical renal outcomes might be associated with the additional effects of blood pressure lowering (Lovshin and Gilbert, 2015). Nevertheless, more researchers considered that was a comprehensive outcome from multiple effects, such as insulin levels, serum uric levels, and body weight (Zhang et al., 2020). In addition, the effects of SGLT2i on renal neurohormonal pathway and arterial stiffness were also considered to be associated with the process (Perrone-Filardi et al., 2017).

The present findings show whether 100 mg or 300 mg of canagliflozin is administered for more than 13 weeks, the changes in eGFR and creatinine are not significant for T2DM patients without chronic kidney disease. This result shows that long-term use of canagliflozin does not have a significant dose-dependent effect on the renal function of T2DM patients. Findings from previous phase III studies of canagliflozin in patients with T2DM suggested that a reduction in eGFR occurs during the early phase of treatment with canagliflozin (the first 4–6 weeks) (Bode et al., 2013; Cefalu et al., 2013; Leiter et al., 2015). Moreover, insights from CREDENCE trial indicated that a drop in eGFR of up to 30% was not an indication to stop canagliflozin as it did not map on to an increase risk of adverse events (Oshima et al., 2021). In the present analysis, we have shown that there is a small decrease in eGFR in patients who are treated with 300 mg/ day canagliflozin follow-up durations more than 13 weeks, but there was a great deal of heterogeneity among the included trials. The observed heterogeneity of the effect on eGFR and creatinine might be the result of disparities in the baseline kidney function of participants in the various trials. However, there is published evidence that treatment with an SGLT2i reduces eGFR in the short term by 3–4 ml/min (Heerspink et al., 2017; Neal et al., 2017). This effect occurs after a single dose, persists with chronic administration, and is quickly reversed after the termination of treatment. The study by Barnett et al. suggested that the treatment of patients with chronic kidney disease stage 3 with empagliflozin decreases eGFR by ∼4 ml/min after 12 weeks, but when drug administration was terminated, the eGFR returned to its baseline level, which implies that the reduction in eGFR is hemodynamic, rather than a result of renal injury in these patients (Barnett et al., 2014).

In the present study, we have performed a comprehensive evaluation of the effects of canagliflozin on cardiovascular and renal outcomes in patients with T2DM. However, there were several limitations to the meta-analysis. First, the inclusion criteria and the definitions of the endpoints differed between the included trials, although these differences were relatively minor. Second, the studies included in the analysis contained relatively few patients with more advanced chronic kidney disease.

## Conclusion

In the present analysis, we have shown that canagliflozin has moderately beneficial effects on cardiovascular and renal outcomes in patients with T2DM. Moreover, we have shown that different dose of canagliflozin has no significant effects on changing the levels of eGFR and serum creatinine for long-term medication.

## Data Availability

The original contributions presented in the study are included in the article/Supplementary Material, further inquiries can be directed to the corresponding author/s.

## Supplementary Material

Supplementary Figure 1 Forest plot of the effect of 100 mg/day canagliflozin on the eGFR of the T2DM after sensitivity analysis of removing one heterogenous trial **(A)** Forest plot of the effect of 300 mg/day canagliflozin on the eGFR of the T2DM participants after sensitivity analysis of removing one heterogenous trial **(B)**.

Supplementary Figure 2 Forest plot of the effect of 100 mg/day canagliflozin on the Creatinine of the T2DM after sensitivity analysis of removing one heterogenous trial **(A)**. Forest plot of the effect of 300 mg/day canagliflozin on the Creatinine of the T2DM participants after sensitivity analysis of removing one heterogenous trial **(B)**.
