# Supplementary figures and images for: Canagliflozin for Prevention of Cardiovascular and Renal Outcomes in type2 Diabetes: A Systematic Review and Meta-analysis of Randomized Controlled Trials
Source: Front Pharmacol. 2021 Jul 19;12:691878. doi: 10.3389/fphar.2021.691878 (PMC8327383; doi:10.3389/fphar.2021.691878)

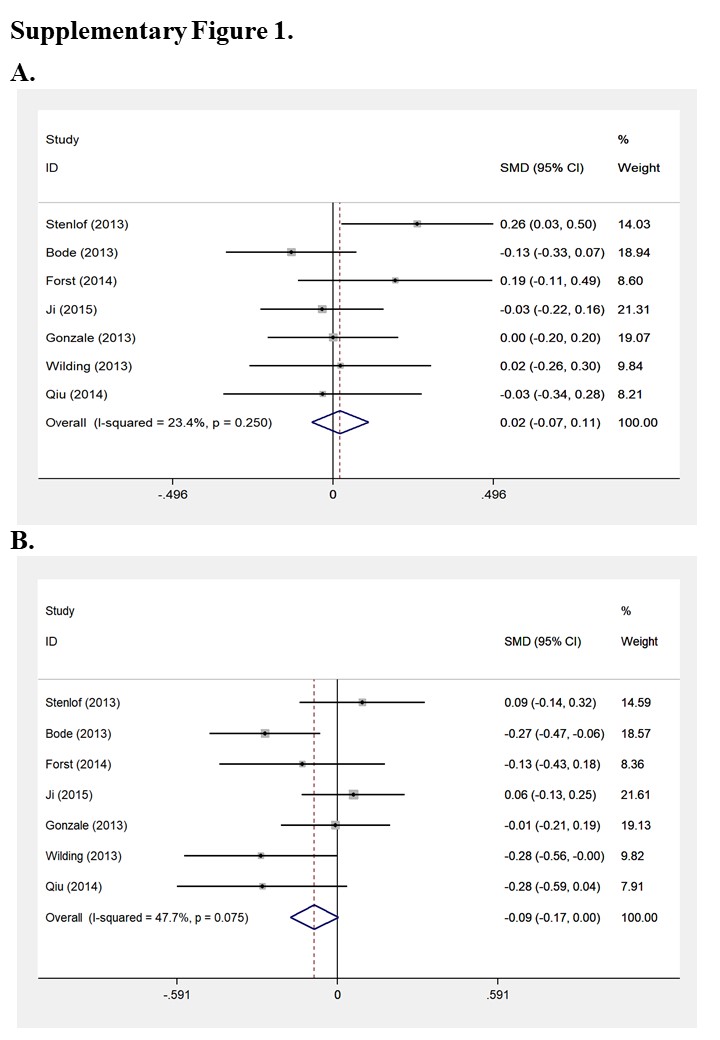

Supplement: Supplementary Figure 1 — Forest plot of the effect of 100 mg/day canagliflozin on the eGFR of the T2DM after sensitivity analysis of removing one heterogenous trial (A) Forest plot of the effect of 300 mg/day canagliflozin on the eGFR of the T2DM participants after sensitivity analysis of removing one heterogenous trial (B). [file Image1.JPEG]

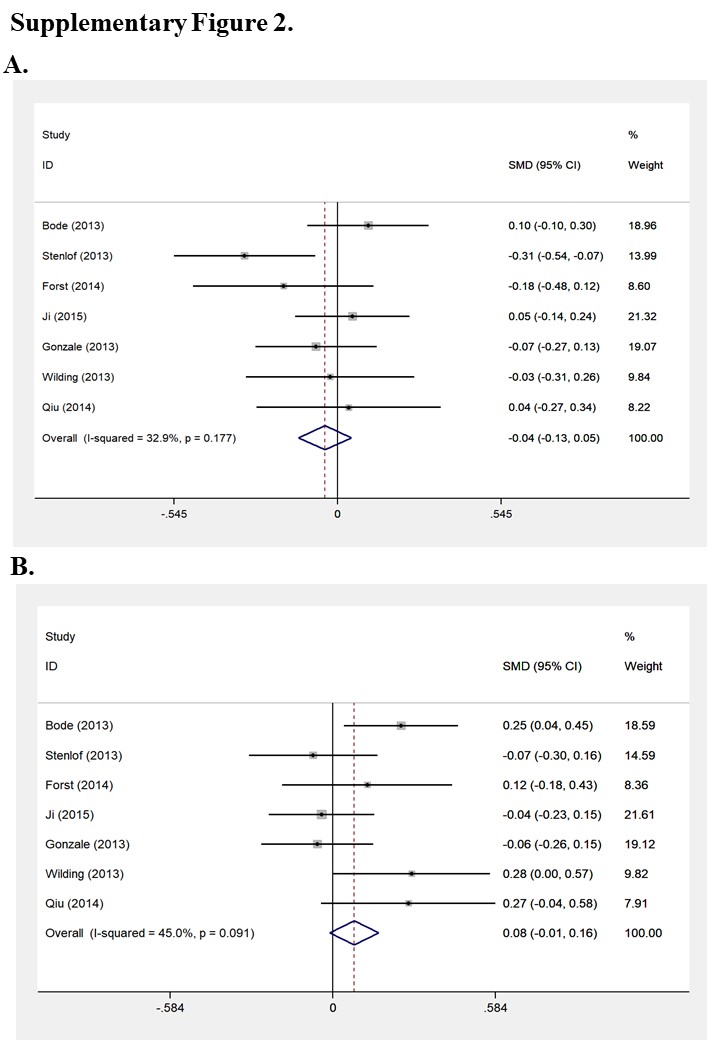

Supplement: Supplementary Figure 2 — Forest plot of the effect of 100 mg/day canagliflozin on the Creatinine of the T2DM after sensitivity analysis of removing one heterogenous trial (A). Forest plot of the effect of 300 mg/day canagliflozin on the Creatinine of the T2DM participants after sensitivity analysis of removing one heterogenous trial (B). [file Image2.JPEG]
